# Speckle tracking imaging for evaluation of effects of positive end-expiratory pressure level on right ventricular function

**DOI:** 10.1186/cc14218

**Published:** 2015-03-16

**Authors:** M Türker, A Pirat, A Camkiran Firat, B Pirat, A Sezgin, G Arslan

**Affiliations:** 1 Baskent University, Ankara, Turkey

## Introduction

Positive end-expiratory pressure (PEEP) is commonly used to correct hypoxemia in the ICU. However, PEEP may impair right ventricular functions by increasing its afterload. Speckle tracking imaging (STI) is a new echocardiography strain analysis technique that provides direct assessment of myocardial contractility during systole and diastole. The aim of this study was to evaluate the effects of different PEEP levels on right ventricular functions by using STI in patients undergoing coronary artery bypass grafting surgery.

## Methods

After ethics committee approval and patients' written consent, we prospectively analyzed 20 CABG surgery patients. After initiation of mechanical ventilation and before sternotomy, 5, 10, and 20 cmH_2_O PEEP were applied in 5-minute intervals consequently. After stabilization at each PEEP level, four-chamber and two-chamber images of the right ventricle were recorded using TEE. The right ventricle diameter, velocity, longitudinal strain, SR, and fractional area change (RVFAC) were calculated and evaluated from the recorded images.

## Results

The mean age of study patients (85% male) was 59.7 ± 10.5 years. Intraoperative mean, systolic, and diastolic arterial blood pressures and heart rate were similar at the three PEEP levels. Compared with 5 and 10 cmH_2_O PEEP, mean RVFAC significantly decreased at 20 cmH_2_O PEEP (*P *= 0.001). Right ventricle velocity reduced with incremental PEEP increases (*P *< 0.05). Mean SR values decreased at 20 cmH_2_O PEEP when compared with 5 cmH_2_O PEEP (*P *= 0.03). Mean right ventricle diameter measurements decreased with incremental PEEP increases; however, this decrease was significantly different between 20 cmH_2_O PEEP and other two PEEP levels (*P *= 0.01). The mean right ventricle strain value significantly decreased at 20 cmH_2_O PEEP when compared with other two PEEP levels (*P *< 0.001 for both).

## Conclusion

Compared with 5 and 10 cmH_2_O PEEP levels, right ventricle functions in terms of strain, SR, right ventricle diameter, and RVFAC were significantly impaired at 20 cmH_2_O PEEP level.

